# A novel differential diagnostic model based on multiple biological parameters for immunoglobulin A nephropathy

**DOI:** 10.1186/1472-6947-12-58

**Published:** 2012-06-27

**Authors:** Jing Gao, Yong Wang, Zhennan Dong, Zhangming Yan, Xingwang Jia, Yaping Tian

**Affiliations:** 1 Department of Clinical Biochemistry, State Key Laboratory of Kidney Disease, Chinese PLA General Hospital, Beijing, 100853, China; 2 Division of Nephropathy, State Key Laboratory of Kidney Disease, Chinese PLA General Hospital, Beijing, 100853, China; 3 School of Life Sciences, Tsinghua University, Beijing, 100084, China

**Keywords:** Primary kidney disease, IgA nephropathy, Multiparameter analysis

## Abstract

**Background:**

Immunoglobulin A nephropathy (IgAN) is the most common form of glomerulonephritis in China. An accurate diagnosis of IgAN is dependent on renal biopsies, and there is lack of non-invasive and practical classification methods for discriminating IgAN from other primary kidney diseases. The objective of this study was to develop a classification model for the auxiliary diagnosis of IgAN using multiparameter analysis with various biological parameters.

**Methods:**

To establish an optimal classification model, 121 cases (58 IgAN vs. 63 non-IgAN) were recruited and statistically analyzed. The model was then validated in another 180 cases.

**Results:**

Of the 57 biological parameters, there were 16 parameters that were significantly different (*P* < 0.05) between IgAN and non-IgAN. The combination of fibrinogen, serum immunoglobulin A level, and manifestation was found to be significant in predicting IgAN. The validation accuracies of the logistic regression and discriminant analysis models were 77.5 and 77.0%, respectively at a predictive probability cut-off of 0.5, and 81.1 and 79.9%, respectively, at a predictive probability cut-off of 0.40. When the predicted probability of the equation containing the combination of fibrinogen, serum IgA level, and manifestation was more than 0.59, a patient had at least an 85.0% probability of having IgAN. When the predicted probability was lower than 0.26, a patient had at least an 88.5% probability of having non-IgAN. The results of the net reclassification improvement certificated serum Immunoglobulin A and fibrinogen had classification power for discriminating IgAN from non-IgAN.

**Conclusions:**

These models possess potential clinical applications in distinguishing IgAN from other primary kidney diseases.

## Background

While some nephrologists may administer tentative drugs to patients with chronic kidney disease (CKD) based on the clinical manifestation prior to performing a renal biopsy, they still depend on a percutaneous renal biopsy to make a definite histological diagnosis, and thereby, determine an efficient drug administration strategy [1-3], especially for patients with resistance or unresponsiveness to immunosuppressive agents, anticoagulants, and/or angiotensin-converting enzyme inhibitor (ACEI) [4-6]. However, despite the advantages of being safe, simple, and easy, this invasive procedure is not risk-free [7]. Furthermore, based on our clinical experiences, at times, renal biopsies cannot be performed on certain patients due to contraindications [8,9], patient refusal, and insufficient operative skills of physicians at certain hospitals. Moreover, the pathologic diagnosis obtained from renal biopsies may be variable. It was previously reported that there was a common change in the histological patterns of lupus nephritis with repeated renal biopsies [10]. This may be due to disease progression, different surgeons performing the biopsy, different biopsy amounts and parts of tissue obtained, and discordant opinions from different nephropathologists [11,12]. Thus, patients would benefit if there was a non-invasive and practical classification model for discriminating the pathological subtypes of kidney disease.

It also has been previously reported that mathematical models may be used to classify different diseases or stages of diseases [13,14]. In fact, some classification equations are already being used in kidney disease. In our previous retrospective study, we reported that the combination of six serum indicators could discriminate immunoglobulin A nephropathy (IgAN) from non-immunoglobulin A nephropathy (non-IgAN) with an 82.3% sensitivity and a 68.6% specificity [15]. This classification method was found to be efficient in the auxiliary diagnosis of IgAN, which is still the most common form of glomerulonephritis in China [16].

In the present study, we utilized common statistical analyses (including logistic regression and discriminant analyses) and typical biological parameters to determine clinically practical classification equations for IgAN and non-IgAN.

## Methods

### Design

The present study was a retrospective cohort study, was conducted in accordance with the Declaration of Helsinki, and approved by the Medical Ethics Committee of the Chinese PLA General Hospital. Patient research consent form was presented as Additional File 1. Fasting blood samples were collected on the second day after patients were admitted into our hospital, according to the established inclusion criteria. Patients were then screened again, according to established exclusion criteria, and divided into two groups, one for establishing a classification model (after 2011), and the other for validating the classification model (before 2011).

### Patients

The inclusion criteria were established to pre-screen all patients. The inclusion criteria were as follows: a) the patient was admitted into the Division of Nephrology at our hospital for the first time; b) a renal biopsy had not been previously performed on the patient for the exact pathologic diagnosis at our or any other hospital; c) the patient was not previously undergoing anti-coagulation, immunosuppression, and/or renal replacement therapy; d) the patient may present with either hepatitis, diabetes, hypertension, or lupus, but not with a tumor; and e) the patient approved to undergo a renal biopsy during the hospital admission. The exclusion criteria used for the final selection of cases were as follows: a) if for any reasons the renal biopsy was not preformed on the included patient (e.g. the patient refused a renal biopsy examination, the patient’s condition worsened during the period of admission, the kidneys of the patient were atrophied or sclerotic.); b) the pathological results indicated that the patient has secondary kidney disease, including diabetic nephropathy, lupus nephritis, hepatitis-related nephropathy; and c) the pathological results could not ascertain whether the patient has primary nephropathy. Based on the exclusion criteria, 301 cases were selected. The immunofluorescence findings, exact histopathological diagnosis for non-IgAN, and Oxford classification score for IgAN of the 121 patients allocated into the ‘modeling’ group, which was used in establishing the classification model, are listed in Additional file 2.

### Samples and blood tests

Blood samples of all included patients underwent blood coagulation testing (STA-R automatic coagulation analyzer, Stago), blood routine examination (Xe-2100 automatic blood analyzer, Sysmex), clinical biochemistry testing (Roche Modular DDP, Roche), immunoglobulin-complement testing (BNΙΙ particular globin analyzer, Siemens), and tumor marker testing (Roche Modular E170, Roche). The remaining sera were preserved at −80 °C.

### Biological parameters and data grouping

Besides “manifestation”, the other 56 biological parameters were listed in Table 1. Data on all of 57 biological parameters were collected and divided into two groups, according to the renal biopsy results: the IgA nephropathy (IgAN) group, which was defined as the presence of IgA immune complex deposits predominantly within the mesangial region of the renal glomerulus, and the non-IgA nephropathy (non-IgAN) group, which was defined as a lack of IgA immune complexes or the absence of IgA immune complex deposits predominantly within the mesangial region of the renal glomerulus. The selected 301 cases were divided into either the ‘modeling’ group (after 2011) or the ‘validation’ group (before 2011).

**Table 1:** Biological parameters assessed in the present study

**Index**	**Full name**	**Abbreviation**	**Reference range**
1	Carcinoembryonic antigen	CEA	0-5 μg/L
2	Alpha fetoprotein	AFP	0-20 μg/L
3	Carbohydrate antigen 125	CA125	0.1-35 u/ml
4	Carbohydrate antigen 199	CA199	0.1-37 u/ml
5	Carbohydrate antigen 153	CA153	0.1-30 u/ml
6	Carbohydrate antigen 724	CA724	0.1-10 u/ml
7	Cytokeratin fragment 21-1	CYFRA21-1	0.1-4 ng/ml
8	Neuron specific enolase	NSE	0-24 ng/ml
9	Squamous cell carcinoma related antigen	SCC	0-1.5 ug/L
10	Glucose	Glu	3.4-6.2 mmol/L
11	Total protein	TP	55-80 g/L
12	Albumin	ALB	35-50 g/L
13	Urea nitrogen	UN	1.8-7.5 mmol/L
14	Creatinine	Cr	30-110 μmol/L
15	Uric acid	Ua	104-444 μmol/L
16	Total cholesterol	CH	3.1-5.7 mmol/L
17	Triglyceride	TG	0.4-1.7 mmol/L
18	High density lipoprotein cholesterol	HDL	1-1.6 mmol/L
19	Low density lipoprotein cholesterol	LDL	0-3.4 mmol/L
20	Potassium	K	3.5-5.5 mmol/L
21	Sodium	Na	130-150 mmol/L
22	Calcium	Ca	2.25-2.75 mmol/L
23	Chloride	Cl	94-110 mmol/L
24	Phosphorus	P	0.97-1.62 mmol/L
25	Magnesium	Mg	0.6-1.4 mmol/L
26	Carbon dioxide	CO2	20.2-30 mmol/L
27	Total bilirubin	TB	0-21 μmol/L
28	Direct bilirubin	DB	0-8.6 μmol/L
29	Alanine aminotransferase	ALT	0-40 U/L
30	Aspartate aminotransferase	AST	0-40 U/L
31	Lactate dehydrogenase	LDH	40-250 U/L
32	Creatine kinase	CK	2-200 U/L
33	γ-Glutamyltransferase	GGT	0-50 U/L
34	Alkaline phosphatase	ALP	0-130 U/L
35	International normalized ratio	INR	0.8-1.2
36	Fibrinogen	FIB	2.0-4.0 g/L
37	Prothrombin time	PT	11.0-15.0 s
38	Prothrombin activity	PA	70-120%
39	Activated coagulation time of whole blood	APTT	30-45 s
40	D-dimer	D2	0.0-0.5 μg/L
41	β2-microglobulin	B_2_MG	0.07-0.18 mg/dl
42	Serum immunoglobulin A	sIgA	70-180 mg/dl
43	Serum immunoglobulin G	sIgG	700-1600 mg/dl
44	Serum immunoglobulin E	sIgE	0-100 IU/ml
45	Serum immunoglobulin M	sIgM	40-230 mg/dl
46	Complement 3	C3	90-180 mg/dl
47	Complement 4	C4	10-40 mg/dl
48	Prealbumin	PA	20-40 mg/dl
49	Red blood count	RBC	Male:4.3-5.9 * 10^12^/L
			Female: 3.9-5.2 * 10^12^/L
50	Hemoglobin	HB	Male: 137-179 g/L
			Female: 116-155 g/L
51	White blood count	WBC	3.5-10 * 10^9^/L
52	Platelet	PLT	100-300 * 10^9^/L
53	Body mass index	BMI	18-25
54	Hypertension	HP	
55	Gender	Gender	
56	Age	Age	

### Statistic analysis

SPSS 17.0 was used for data analysis. Statistical analyses, including t-tests, nonparametric tests (i.e. Mann–Whitney U-test), chi-square test and bivariate correlation tests, were conducted for the selection of different parameters. Logistic regression and discriminant analyses were used in establishing the classification model for IgAN and non-IgAN.

The net reclassification improvement (NRI) was used for evaluating the classification improvement of the biological parameters.

## Results

### Patient characteristics

The ‘modeling’ group consisted of 121 cases, including 58 IgAN and 63 non-IgAN cases (average age of 35.6 ± 12.4 and 39.7 ± 15.3 years, respectively). The ‘validation’ group consisted of 180 cases, including 93 IgAN and 87 non-IgAN cases (average age of 32.8 ± 11.6 and 43.7 ± 15.7 years, respectively). Patient characteristics of the ‘modeling’ and ‘validation’ groups are presented in Table 2.

**Table 2:** Patients characteristics of the modeling and validation groups

	**Modeling group**		**Validation group**	
	IgAN	Non-IgAN	IgAN	Non-IgAN
Number	58	63	93	87
Age (years)	35.6 ± 12.4	39.7 ± 15.3	32.8 ± 11.6 ^b^	43.7 ± 15.7 ^b^
Male : female	39:19	33:30	53:40	48:39
Hypertension (%)	28 (48.3%)	28 (44.4%)	45 (48.9%)	50 (57.5%)
Body mass index (kg/m^2^)	24.9 ± 3.7	24.8 ± 4.0	24.1 ± 4.2	25.2 ± 4.3
***Manifestation***				
Chronic nephritis syndrome (%)	44 (75.9%)^a^	23 (36.5%)^a^	75 (80.6%)^a^	31 (35.6%)^a^
Nephrotic syndrome (%)	10 (17.2%)^a^	33 (52.4%)^a^	10 (10.8%)^a^	51 (58.6%)^a^
Isolated proteinuria or hematuria (%)	4 (6.9%)^a^	7 (11.1%)^a^	8 (8.6%)^a^	5 (5.7%)^a^
***Renal function***				
Normal (%)	43 (74.1%)	48 (76.2%)	67 (72.0%)	69 (79.3%)
Chronic renal insufficiency (%)	15 (25.9%)	11 (17.5%)	25 (26.9%)	14 (16.1%)
Acute renal insufficiency (%)	0	4 (6.3%)	1 (1.1%)	4 (4.6%)

^a^*P* < 0.05 via Chi-square test, between IgAN and Non-IgAN; ^b^*P* < 0.05 via *t*-test, between IgAN and Non-IgAN.

***Notes:*** Hypertension was defined as systolic blood pressure (BP) ≥140 mmHg, diastolic BP ≥90 mmHg, or use of antihypertensive medications. Chronic nephritis syndrome was defined as proteinuria or hematuria with hypertension or edema. Nephrotic syndrome was defined as persistent proteinuria of more than 3.5 g/1.73 m^2^/24 h, hypoalbuminemia or albumin levels ≤30 g/L, edema, and varying degrees of hyperlipidemia. Isolated proteinuria or hematuria was defined as a urine protein excretion >0.3 g/1.73 m^2^/24 h or urine red blood cell (RBC) >3/HP with normal renal function and without hypertension and edema. Normal renal function was defined as estimated glomerular filtration rate (GFR) >90 ml/min/1.73 m^2^ on at least two occasions. Chronic renal insufficiency was defined as an estimated GFR <60 ml/min/1.73 m^2^ on at least two occasions with chronic kidney disease. Acute renal insufficiency was defined as an abrupt (within 48 h) reduction in kidney function, according to the Acute Kidney Injury Network (AKIN) criteria.

***Abbreviations:****IgAN*, Immunoglobulin A nephropathy; *Non-IgAN*, non-immunoglobulin A nephropathy.

### Univariate analysis

T- and Mann–Whitney U-tests were performed to determine significant differences in all of the 57 parameters studied between the IgAN and non-IgAN groups. The mean ± SD, median with extremes, and the *P*-values are presented in Additional file 3. Besides manifestation, there were 15 serological indicators that were significantly different (*P* < 0.05) between IgAN and non-IgAN (Table 3). Some of these parameters, including serum fibrinogen (FIB), serum D-dimer (D2), serum immunoglobulin A (sIgA), serum immunoglobulin G (sIgG), serum albumin (ALB), serum total protein (TP), serum total cholesterol (CH), serum low density lipoprotein (LDL), serum triglyceride (TG), and serum urea (UN), have been previously implicated in kidney disease [17]. However, serum direct bilirubin (DB), serum calcium (Ca), serum alkaline phosphatase (ALP), serum carbohydrate antigen 19–9 (CA199), and serum carbohydrate antigen 15–3 (CA153) have never been implicated in kidney disease.

**Table 3:** Differences in the serological parameters between IgAN and non-IgAN

**Parameter**	**Mean ± SD**		***P*-value**
	**IgAN**	**Non-IgAN**	
FIB^a^	3.63 ± 1.00	5.00 ± 2.60	0.000
sIgG^a^	1018.5 ± 307.0	858.2 ± 352.7	0.020
TP ^b^	66.2 ± 9.5	57.3 ± 12.7	0.000
ALB ^b^	39.1 ± 6.5	31.7 ± 9.7	0.000
Ca ^b^	2.22 ± 0.15	2.09 ± 0.18	0.000
D2^a^	0.70 ± 1.26	1.46 ± 2.99	0.019
sIgA ^a^	331.3 ± 103.9	241.5 ± 102.3	0.000
CH^a^	4.84 ± 1.24	6.38 ± 2.79	0.002
DB^a^	3.1 ± 1.8	2.4 ± 1.3	0.029
LDL^a^	2.98 ± 1.00	4.14 ± 2.23	0.003
CA153^a^	11.9 ± 5.2	14.9 ± 7.7	0.038
TG^a^	1.7 ± 1.1	2.1 ± 1.0	0.013
ALP^a^	68.8 ± 40.2	81.0 ± 50.3	0.015
CA199^a^	12.0 ± 8.7	18.8 ± 20.3	0.046
UN ^a^	6.6 ± 3.1	6.1 ± 3.4	0.048

^a^*P* < 0.05 via Mann–Whitney *U*-test; ^b^*P* < 0.05 via *t*-test

***Abbreviations:****FIB*, fibrinogen; *sIgG*, serum immunoglobulin G; *TP*, total protein; *ALB*, albumin; *Ca*, calcium; *D2*, D-dimer; *sIgA*, serum immunoglobulin A; *CH*, total cholesterol; *DB*, direct bilirubin; *LDL*, low density lipoprotein; *CA153*, carbohydrate antigen 15–3; *TG*, triglyceride; *ALP*, alkaline phosphatase; *CA199*, carbohydrate antigen; *UN*, urea.

**Figure 1:**
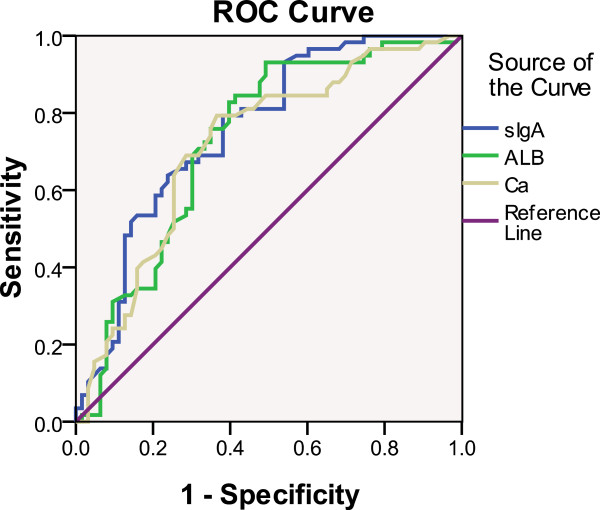
**Respective ROC Curve of sIgA, ALB and Ca between IgAN and non-IgAN.** ROC curves for serum immunoglobulin A level (sIgA), ALB (albumin) and Ca (calcium) in immunoglobulin A nephropathy (IgAN) and non-immunoglobulin A nephropathy (non-IgAN) patients. The state variable is IgAN.

Receiver operating characteristics (ROC) curve analyses were performed on these 57 parameters, and the findings (i.e. area under curve (AUC), 95% confidence interval (CI) and *P*-value) were presented in Additional file 4. Table 4 contained the C statistics of 16 significantly different serological parameters, among which five parameters, specifically TP, ALB, Ca, FIB, and sIgA, with the additional manifestation were highly significant variables (*P* < 0.01). sIgA, ALB, and Ca had the top three diagnostic levels (i.e. 75.6, 72.7, and 71.8%) between IgAN and non-IgAN (Figure 1).

**Table 4:** C statistics of the ROC curves for the 16 significant parameters between IgAN and non-IgAN

**Parameter**	**AUC**	**95% Confidence lnterval**		***P*-value**
		**lower Bound**	**upper Bound**	
CA199 ^b^	0.605	0.505	0.706	0.046
CA153 ^b^	0.609	0.509	0.709	0.038
TP ^a^	0.703	0.611	0.796	0.000
ALB ^a^	0.727	0.636	0.819	0.000
UN ^a^	0.604	0.503	0.706	0.048
CH ^b^	0.667	0.572	0.763	0.002
TG ^b^	0.631	0.532	0.731	0.013
LDL ^b^	0.657	0.560	0.753	0.003
Ca ^a^	0.718	0.625	0.810	0.000
Cl ^b^	0.628	0.528	0.729	0.015
DB ^a^	0.615	0.514	0.717	0.029
ALP ^b^	0.628	0.528	0.729	0.015
FIB ^b^	0.712	0.621	0.804	0.000
D2 ^b^	0.626	0.525	0.727	0.019
sIgA ^a^	0.756	0.670	0.842	0.000
sIgG ^a^	0.623	0.524	0.722	0.020

***Abbreviations:****CA199*, carbohydrate antigen; *CA153*, carbohydrate antigen 15–3; *TP*, total protein; *ALB*, albumin; *UN*, urea; *CH*, total cholesterol; *TG*, triglyceride; *LDL*, low density lipoprotein; *Ca*, calcium; *Cl*, chloride; *DB*, direct bilirubin; *ALP*, alkaline phosphatase; *FIB*, fibrinogen; *D2*, D-dimer; *sIgA*, serum immunoglobulin A; *sIgG*, serum immunoglobulin G.

Based on the findings of the t- or U-tests and ROC curve analyses, 16 parameters, including manifestation, sIgA, sIgG, D2, TP, ALB, CH, TG, LDL, UN, DB, Ca, ALP, CA199, and CA153, were selected for further analysis.

### Correlation analysis of pre-selected parameters

**Figure 2:**
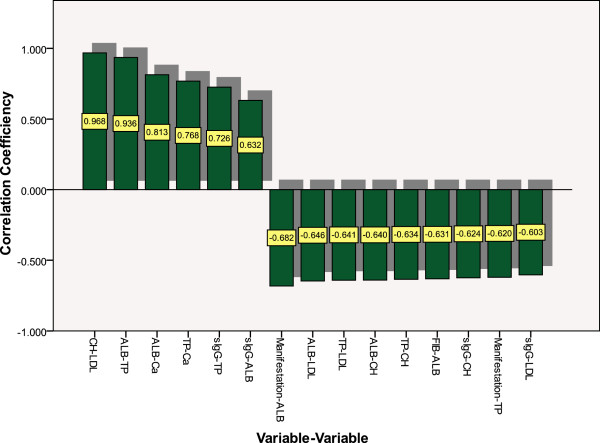
Correlation coefficients between two variables of pre-selected variables.

Multiple correlations were found among biological parameters or medical data. However, multiparameter analysis requires that each explanatory variable is independent. Thus, bivariate correlation tests were executed to eliminate parameters with a high multicollinearity before performing multiparameter analysis. It was found that there were significant correlations (*P* < 0.01) among almost half of the 16 parameters, specifically among “manifestation”, FIB, sIgG, TP, ALB, CH, LDL, and Ca (Figure 2). Based on our clinical experience, we removed TP, LDL, and Ca, and selected the other 13 parameters for further analysis.

### Modeling with multiparameter analysis

Logistic regression and discriminant analyses were used to establish the IgAN and non-IgAN classification model. The 13 pre-selected parameters were manifestation, FIB, D2, sIgA, sIgG, ALB, UN, CH, TG, DB, ALP, CA199, and CA153.

**Figure 3:**
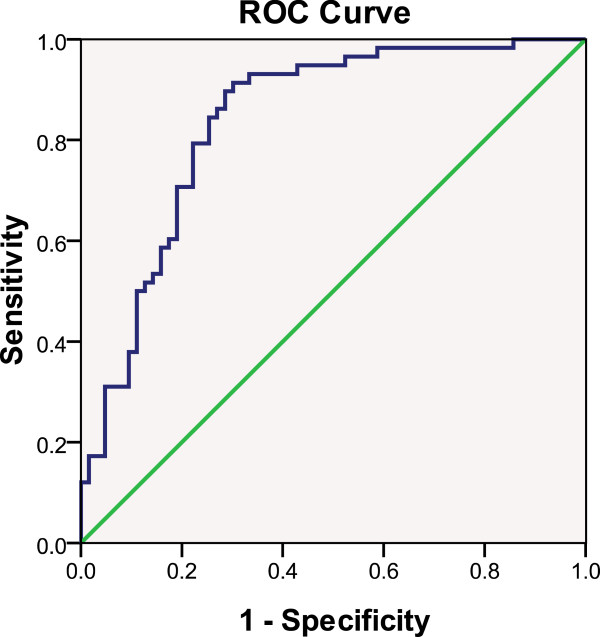
**Figure**3**Area under ROC curve of the predicted probability of IgAN with “FIB + sIgA + Manifestation” combination from logistic regression.** Area under the ROC curve for predicting immunoglobulin A nephropathy (IgAN) with the equation derived via logistic regression analysis, which includes the “fibrinogen (FIB) + serum immunoglobulin A level (sIgA) + manifestation” combination. The state variable is IgAN.

a) Model based on logistic regression analysis: Except manifestation, the other 12 pre-selected parameters were substituted into a binary logistic regression as an explanatory variable via the “Enter” method of a univariate analysis (Table 5). Parameters that had a *P* < 0.2 in univariate logistic regression were chosen to prevent the exclusion of important variables. With the exception of UN, the other 12 variables had a *P* < 0.2 and were all substituted into the multivariate logistic regression, using the forward conditional method of entry. The predicted probabilities (PRE-1) were calculated and saved. Using multivariate logistic regression analysis, it was found that only manifestation, FIB, and sIgA were significant predictors of IgAN (Table 6). The classification model with these 3 parameters was evaluated, and it was found that accuracy was 76.9%, sensitivity was 74.1%, specificity was 79.4%, false positive rate (α) was 20.6%, false negative rate (β) was 25.9%, positive predictive value (PPV) was 76.8%, negative predictive value (NPV) was 76.9%, positive likelihood ratio (+LR) was 3.59, negative likelihood ratio (−LR) was 0.32, and Youden's index was 0.535. The area under the ROC curve with PRE-1 for IgAN was 83.8% (*P* < 0.0001, 95%CI: 0.766-0.910) (Figure 3).

**Table 5:** Univariate logistic regression analysis of the 12 pre-selected serological parameters

**Parameter**	**B**	**S.E.**	**Wald**	**df**	**Sig.**	**Exp(B)**
FIB	2.485	0.735	11.432	1	0.001	0.534
D2	−0.242	0.161	2.275	1	0.132	0.785
sIgA	0.009	0.002	16.440	1	0.000	1.009
sIgG	0.001	0.001	6.396	1	0.011	1.001
UN	0.049	0.057	0.740	1	0.390	1.051
ALB	0.109	0.026	17.498	1	0.000	1.115
TG	−0.326	0.186	3.068	1	0.080	0.722
CH	−0.429	0.131	10.673	1	0.001	0.651
DB	0.308	0.126	5.970	1	0.015	1.361
ALP	−0.007	0.005	1.883	1	0.170	0.993
CA199	−0.041	0.019	4.855	1	0.028	0.960
CA153	−0.071	0.031	5.293	1	0.021	0.931

***Abbreviations:****FIB*, fibrinogen; *D2*, D-dimer; *sIgA*, serum immunoglobulin A; *sIgG*, serum immunoglobulin G; *UN*, urea; *ALB*, albumin; *TG*, triglyceride; *CH*, total cholesterol; *DB*, direct bilirubin; *ALP*, alkaline phosphatase; *CA199*, carbohydrate antigen; *CA153*, carbohydrate antigen 15–3.

**Table 6:** Parameters used in the multivariate logistic regression analysis for developing the classification model

**Parameter**	**B**	**S.E.**	**Wald**	**df**	**Sig.**	**Exp(B)**	**95% CI for EXP(B)**	
							**Lower**	**Upper**
Manifestation	−1.089	0.423	6.637	1	0.010	0.336	0.147	0.771
FIB	−0.326	0.165	3.918	1	0.048	0.722	0.522	0.997
sIgA	0.011	0.003	16.724	1	0.000	1.011	1.006	1.016
Constant	−0.648	0.858	0.569	1	0.450	0.523		

***Abbreviations:****FIB*, fibrinogen; *sIgA*, serum immunoglobulin A.

The “FIB + sIgA + Manifestation” combination was significant in the classification of IgAN and non-IgAN, as determined via logistic regression analysis. The classification equation, which includes these 3 parameters, for predicting IgAN is as follows:

(1) $$\text{PRE}-1\;=1-\;\frac{1}{\left[1+{\text{e}}^{\left(-0.648–0.326\text{FIB}+0.011\text{sIgA}-1.089\text{Manifestation}\right)}\right]}$$

**Figure 4:**
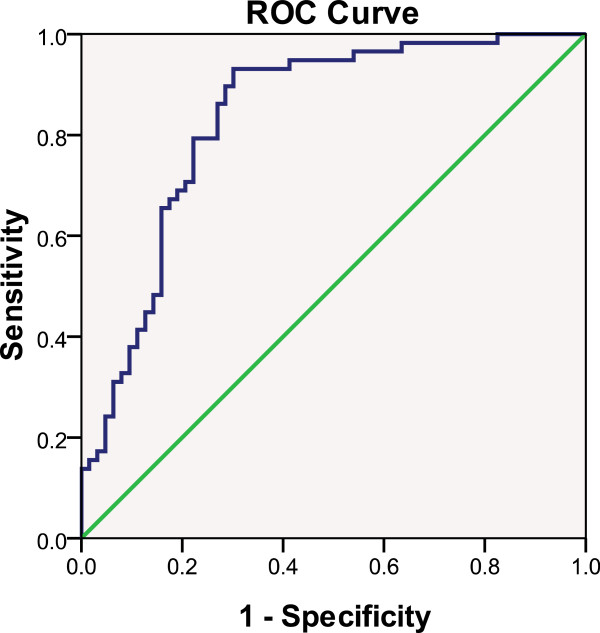
**Area under ROC curve of the predicted probability of IgAN with “FIB + sIgA + Manifestation” combination from discriminant analysis.** Area under the ROC curve for predicting immunoglobulin A nephropathy (IgAN) with the equation derived via discriminant analysis, which includes the “fibrinogen (FIB) + serum immunoglobulin A level (sIgA) + manifestation” combination. The state variable is IgAN.

b) Model based on discriminant analysis: The 13 pre-selected parameters were substituted into a step discriminant analysis. The predicted probabilities (PRE-2) were calculated and saved. Similar to the logistic regression analysis, only sIgA, manifestation, and FIB were significant in the classification of IgAN and non-IgAN (Table 7). The classification model with these 3 parameters was evaluated, and it was found that accuracy was 76.9%, sensitivity was 79.3%, specificity was 74.6%, false positive rate (α) was 25.4%, false negative rate (β) was 20.7%, positive predictive value (PPV) was 74.2%, negative predictive value (NPV) was 79.7%, positive likelihood ratio (+LR) was 3.12, negative likelihood ratio (−LR) was 0.28, and Youden's index was 0.461. The area under the ROC curve with PRE-2 for IgAN was 83.5% (*P* < 0.0001, 95%CI: 0.762-0.909) (Figure 4).

**Table 7:** Parameters used in the discriminant analysis for developing the classification model

**Variables in the analysis**				
Step		Tolerance	Sig. of F to Remove	Wilks' Lambda
1	ALB	1.000	0.000	
2	ALB	1.000	0.000	0.839
	sIgA	1.000	0.000	0.834
3	ALB	0.601	0.123	0.702
	sIgA	0.974	0.000	0.810
	Manifestation	0.593	0.020	0.722
4	sIgA	0.986	0.000	0.846
	Manifestation	0.986	0.000	0.839
5	sIgA	0.967	0.000	0.829
	Manifestation	0.803	0.003	0.733
	FIB	0.789	0.045	0.702

***Abbreviations:****ALB*, albumin; *sIgA*, serum immunoglobulin A; *FIB*, fibrinogen.

The classification equation, which includes the combination of “sIgA + Manifestation + FIB” for predicting IgAN, is as follows:

(2) $$\frac{\text{PRE}-2={\text{e}}^{\left(1.2234\text{Manifestation}+0.028\text{sIgA}+0.463\text{FIB}-6.896\right)}}{\left[{\text{e}}^{\left(1.2234\text{Manifestation}+0.028\text{sIgA}+0.463\text{FIB}-6.896\right)}+{\text{e}}^{\left(2.452\text{Manifestation}+0.018\text{sIgA}+0.713\text{FIB}-6.371\right)}\right]}$$

### Validation of the two models

**Figure 5:**
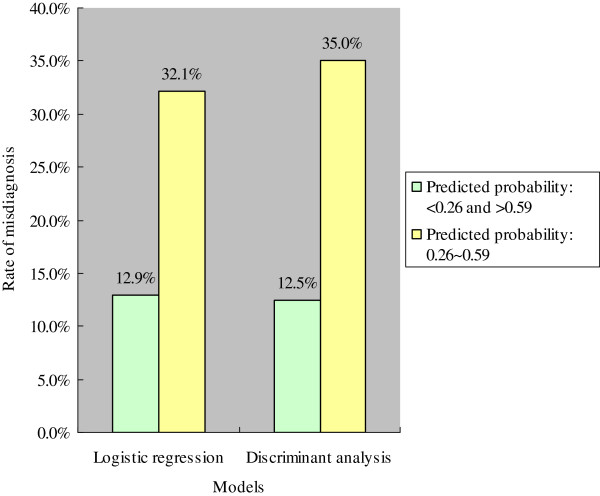
Misdiagnosis rates of the two models with different cut-offs for the predicted probability.

One-hundred and eighty new cases were substituted into the two equations of PRE-1 and PRE-2. Each predicted probability was calculated and compared with the biopsy diagnosis. The sensitivity and specificity were compared between the different cut-off points of predicted probabilities (Table 8). When the cut-off point of the predicted probabilities was decreased to 0.40, the sensitivities of the two models increased, whereas the specificities decreased. When the cut-off point of the predicted probabilities was 0.40, the frequency of misdiagnosis of the two models was higher between 0.26-0.59 than for <0.26 and >0.59 (Figure 5). This indicates that when we use a mathematical model for predicting a clinical diagnosis, we have to pay close attention to the cases near the cut-off points of the predicted probabilities, as they are prone to misdiagnosis. Further analysis indicated that, when the predicted probability is >0.59 or <0.26, the patient has at least an 85.0 or 88.5% probability of having IgAN or non-IgAN, respectively (Table 9).

**Table 8:** Comparison of the diagnostic efficiency of the two models for predicting IgAN and non-IgAN

**Model**	**Cut-off point of of predicted probability**	**Predicted membership**	**Biopsy diagnosis**		**Sensitivity**	**Specificity**	**Accuracy**
			**IgAN**	**Non-IgAN**			
Logistic regression model PRE-1	0.50	IgAN	64	12	68.8%	86.2%	77.5%
		Non-IgAN	29	75			
	0.40	IgAN	77	18	82.8%	79.3%	81.1%
		Non-IgAN	16	69			
Discriminant analysis model PRE-2	0.50	IgAN	64	13	68.8%	85.1%	77.0%
		Non-IgAN	29	74			
	0.40	IgAN	77	20	82.8%	77.0%	79.9%
		Non-IgAN	16	67			

***Abbreviations:****IgAN*, immunoglobulin A nephropathy; *Non-IgAN*, non-immunoglobulin A nephropathy; *PRE*, predicted probability.

**Table 9:** Diagnostic efficiency of the two models when the predicted probabilities is either >0.59 or <0.26 (the cut-off point = 0.4)

**Predicted membership of the models**	**IgAN**	**Non-IgAN**	**Sensitivity**	**Specificity**
***Logistic regression model PRE-1***				
IgAN	54	7	85.7%	88.5%
Non-IgAN	9	54		
***Discriminant analysis model PRE-2***				
IgAN	51	6	85.0%	90.0%
Non-IgAN	9	54		

***Abbreviations:****IgAN*, immunoglobulin A nephropathy; *non-IgAN*, non-immunoglobulin A nephropathy; *PRE*, Predicted probability.

### Analysis of the net reclassification improvement (NRI)

A logistic regression model and a discriminant analysis model were made as two primary models with the parameters of “gender” and “manifestation”. The 12 pre-selected biological parameters (sIgA, ALB, FIB, CH, TG, ALP, D2, sIgG, DB, CA153, CA199 and UN) were put into the algorithm of the net reclassification improvement (NRI) for assessing the classification power between IgAN and non-IgAN. According to above results, we set the predicted probability into four categories: 0 ~ 0.26, 0.26 ~ 0.4, 0.4 ~ 0.59 and 0.59 ~ 1. First, make gender and manifestation into the original parameters of the models. Next, add the other 12 parameters one by one in order of the significance (Table 4) and then check the NRI and *P* value. The results showed that only sIgA and FIB significantly improved the performance of the models. The NRI of sIgA and FIB was 0.290 and 0.168 (*P* < 0.005) in the linear logistic regression model, and was 0.308 and 0.169 (*P* < 0.005) in the linear discriminant analysis model (Table 10). Each step of adding the 12 parameters into the basic models were listed in Additional file 5.

**Table 10:** Net reclassification improvement of the 12 pre-selected biological parameters

**Order**	**Parameter**	**Logistic regression model**		**Discriminant analysis model**	
		**NRI**	***P*-value**	**NRI**	***P*-value**
1	sIgA	0.290	0.001	0.308	0.000
2	ALB	0.023	0.157	0.000	--
3	FIB	0.168	0.003	0.169	0.001
4	CH	-0.022	0.334	-0.034	0.184
5	TG	-0.108	0.037	-0.130	0.017
6	ALP	-0.012	0.762	0.019	0.640
7	D2	-0.011	0.317	0.011	0.581
8	sIgG	-0.022	0.726	0.003	0.962
9	DB	0.022	0.157	-0.011	0.646
10	CA153	0.001	0.985	-0.022	0.593
11	CA199	-0.011	0.317	0.011	0.775
12	sUN	-0.024	0.384	0.022	0.334

***Abbreviations:****NRI*, net reclassification improvement; *sIgA*, serum immunoglobulin A; *ALB*, albumin; *FIB*, fibrinogen; *CH*, total cholesterol; *TG*, triglyceride; *ALP*, alkaline phosphatase; *D2*, D-dimer; *sIgG*, serum immunoglobulin G; *DB*, direct bilirubin; *CA153*, carbohydrate antigen 15–3; *CA199*, carbohydrate antigen; *UN*, urea.

### Decision procedure

**Figure 6:**
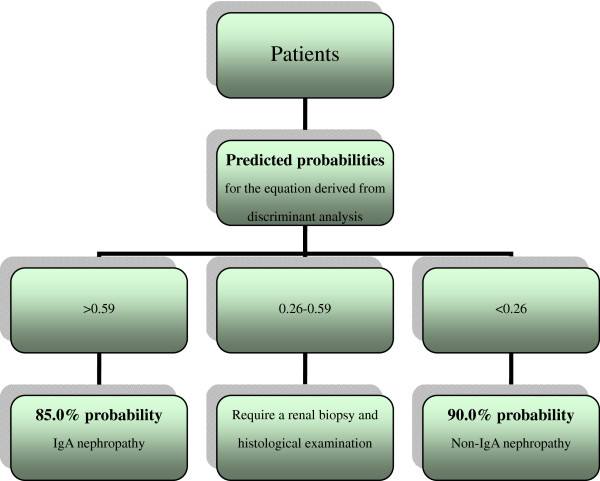
Decision procedure for the diagnosis of immunoglobulin A (IgA) and non-IgA nephropathy in patients with suspected kidney disease.

The decision procedure for the diagnosis of IgA nephropathy in patients with suspected kidney disease, which is based on the validation dataset and the equation from the discriminant analysis, is presented in (Figure 6).

## Discussion

When statistics are used to determine the significant predictors for a diagnosis or classification of a disease, different statistical algorithms, biological datasets, and parameters may result in different outputs [18-20]. Furthermore, multicollinearity is almost always present with medical laboratory parameters, which may also bring out variability and instability in a statistical model [21]. Thus, choosing appropriate variables for multiparameter analysis is very important.

The present study was designed as a cohort study, and was based on a previous retrospective study [15]. Compared with the previous study, this study had more parameters, including fibrinogen, D-dimer, serum IgA, and complement C3, all of which are known biomarkers of kidney diseases [22,23]. Based on univariate analysis, correlation analysis, and clinical experience, 13 out of 57 routine and useful parameters were selected as predictors of IgAN. These were as follows: manifestation, FIB, D2, sIgA, sIgG, UN, ALB, TG, CH, DB, ALP, CA199, and CA153. Three indicators, specifically TP, LDL, and Ca, were screened out, as they demonstrated the highest correlations with the other two indicators (correlation coefficients: TP/ALB = 0.936, LDL/CH = 0.968 and Ca/ALB = 0.813). Similar results were obtained with two of the most frequently-used multiparameter analyses, in particular logistic regression and discriminant analyses, indicating that these three parameters are truly significant in classifying IgAN and non-IgAN.

Furthermore, 180 new cases were used to validate the two equations derived equations for classifying IgAN. The discerning power of the two classification equations was similar in the validation cases. The different cut-off points of the predicted probabilities resulted in different diagnostic efficiencies, indicating that the cases near the cut-off point require more attention. Further analysis indicated that the misdiagnosis rate of cases with predicted probabilities between 0.26-0.59 was higher than of those with predicted probabilities of <0.25 and >0.59 (the cut-off point = 0.4). These results are very interesting and important, as: a) if the predicted probability of a patient is between 0.26-0.59, then the patient needs more testing for diagnosis, such as a renal biopsy; b) if the predicted probability of a patient is >0.59, then the patient has at least an 85.0% possibility of IgAN; and c) if the predicted probability of a patient is <0.26, then the patient has at least an 88.5% possibility of non-IgAN.

The net reclassification improvement (NRI), produced by Penica et al., is used for evaluating the classification improvement when a new marker is put into a primary model [24]. For further investigating the classification power of the pre-selected biological parameters, we used “gender” and “manifestation” to create a basic linear logistic regression model and a linear discriminant analysis model. The results of NRI indicated only sIgA and FIB were positive for discriminating IgAN from non-IgAN in this dataset (Table 10).

The exact pathogenesis of IgAN has not been elucidated up to now. Aberrant IgA1 molecular with the glycans (galactose or sialic acid) deficiencies in the hinge region in circulation is deemed generally to be a crucial and initial factor for the development and pathological characteristics of IgAN [25-28]. The previous reports indicated that abnormally glycosylated IgA1 molecular had more affinity with the specific IgA1 receptor in the mesangial cells [29], was apt to deposit in kidneys combined with circulating IgG molecular or self-assembled macromolecular [30,31], and was hard to clear by liver [32]. Since IgA1 is a predominant isotype of IgA in circulation [33], serum IgA level could reflect serum IgA1 level. Some reports showed that patients with IgAN had elevated serum IgA levels, and consequently, it might be used as a potential diagnostic marker for IgAN [34,35]. Nevertheless, the method by using varying degrees of serum IgA level to make a differential diagnosis for discriminating IgAN from other subtypes of kidney disease is not widely accepted. The present study indicated serum IgA level elevated in patients with both IgAN (331.3 ± 103.9 mg/dl) and non-IgAN (241.5 ± 102.3 mg/dl) according to the reference range 70 ~ 180 mg/dl (Table 1). Serum IgA, seemed like not a specific marker for IgAN, still had significant difference and differentially diagnostic value (area under curve of ROC curve: 75.6%, *P* < 0.0001), which corroborated the views of some previously study [23].

When serum IgA was combined with the other 2 parameters, particularly manifestation and fibrinogen, the diagnostic accuracy of serum IgA increased from 75.6 to 83.9%, as determined by ROC curve analysis, suggesting that, with the exception of serum IgA, clotting mechanisms might be different in the development of IgAN and non-IgAN, which reflected in the proportion of nephrotic syndrome in IgAN (17.2%) and non-IgAN (52.4%). To be precise, serum IgA was a relatively specific marker for IgAN, however fibrinogen and manifestation were two relatively specific markers for non-IgAN. In 63 non-IgAN of the modeling group, 55.6% patients were with membranous nephropathy or minor change disease (Additional file 1). Nephrotic syndrome is the most common clinical manifestation of these two subtypes of glomerular disease [36]. Patients with nephrotic syndrome are always in a state of hypercoagulability and hyperfibrinolysis [37,38], which could be caused by the increased synthesis of blood coagulation factors in liver, the increased consumption of antithrombin, and the decreased levels of protein S, protein C and plasminogen [39,40]. Therefore, as Factor I, serum fibrinogen level was higher in non-IgAN characterized by the predominance of nephrotic syndrome than in IgAN, and accordingly had discerning power between the two groups, as well as D-dimer.

Other significantly different biological parameters between IgAN and non-IgAN, such as TP, ALB, CH, TG, LDL and sIgG, were also linked to the different proportion of nephrotic syndrome (Table 2), which is characterized by mass proteinuria, hypoalbuminemia, edema, and varying degrees of hyperlipidemia [36]. Moreover, given that Ca combines with ALB in blood [41], non-IgAN patients that appeared to have nephrotic syndrome demonstrated decreases in serum levels of Ca after a decrease in ALB. This was confirmed by the high correlation coefficient between Ca and ALB (0.813) in our analysis.

Furthermore, though DB was significantly different between IgAN and non-IgAN, the disparity of the averages was little (3.1 ± 1.8 μmol/L vs. 2.4 ± 1.3 μmol/L), and DB levels in most patients were normal. It is reported serum DB correlated with estimated glomerular filtration rate (eGFR) [42], however, we did not find this correlation with eGFR calculated by the CKD-EPI equation [43] (*P* = 0.35, correlation coefficient = 0.086) in this study. So, we believed the difference of DB between IgAN and non-IgAN had no clinical significance.

We have carried out a study for analyzing the clinical significance of serum CA125 and CA199 levels and their correlation factors in patients with chronic nephropathy, and the results indicated when patients with chronic nephropathy complicated with serous effusions or other factors favoring the formation of serous effusions, such as nephrotic syndrome, serum levels of CA125 and CA199 were apt to increase [44]. And CA153 were also correlated with ALB (correlation coefficient = −0.436, *P* < 0.0001), CH (correlation coefficient = 0.451, *P* < 0.0001), nephrotic syndrome (correlation coefficient = 0.418, *P* < 0.0001), FIB (correlation coefficient = 0.393, *P* < 0.0001) and LDL (correlation coefficient = 0.440, *P* < 0.0001). Thus, these two parameters having significant difference between IgAN and non-IgAN could also due to the different proportion of nephrotic syndrome.

## Conclusions

In the present study, we report on 3 parameters and 2 classification equations that can be used for discriminating between IgAN and non-IgAN with more than 79.9% accuracy. More importantly, when the predicted probability is more than 0.59, a patient has at least an 85.0% probability of having IgAN. However, when the predicted probability is below 0.26, a patient has at least an 88.5% probability of having non-IgAN. These equations may have clinical applicability and value in diagnosing IgAN, and are based on multiparameter analyses with various relevant biological parameters.

## Abbreviations

FIB, Fibrinogen; D2, d-dimer; sIgA, Serum immunoglobulin A; sIgG, Serum immunoglobulin B; ALB, Albumin; TP, Total protein; CH, Total cholesterol; LDL, Low density lipoprotein; TG, Triglyceride; UN, Urea; DB, Direct bilirubin; Ca, Calcium; ALP, Alkaline phosphatase; CA199, Carbohydrate antigen; CA153, Carbohydrate antigen 15–3; CKD, Chronic kidney disease; GFR, Glomerular filtration rate; IgAN, Immunoglobulin A nephropathy; Non-IgAN, Non-immunoglobulin A nephropathy; NRI, Net reclassification improvement.

## Competing interests

The authors have no competing interests to declare.

## Authors’ contributions

All of the authors read and approved the final manuscript. GJ was in charge of testing the parameters, analyzing the data, and preparing the first draft of the manuscript; WY established the inclusion and exclusion criteria for the study, and determined the study groups to which the selected cases belonged to, according to the histopathologic diagnosis; DZN wrote and completed the first draft of this manuscript; YZM completed the analysis of the net reclassification improvement; JXW collected the cases and samples; TYP planned and designed the study. All authors read and approved the final manuscript.

## Authors’ information

GJ is the Technician-in-Charge in the Department of Clinical Biochemistry at the Chinese PLA General Hospital, and is currently completing a doctoral degree. WY is a Nephrologist and Associate Professor in the Division of Nephropathy at the Chinese PLA General Hospital. He possesses profound expertise in chronic kidney disease, and grouped all of the selected cases for this study. DZN is a Senior Technician and Associate Director of the Department of Clinical Biochemistry at the Chinese PLA General Hospital. He has been engaging in clinical laboratory examination work for 30 years. YZM is a PhD candidate in Bioinformatics and Systems Biology at Tsinghua University. JXW has a PhD in the field of diagnostics within the clinical laboratory, and a Senior Technician-in-Charge in the Department of Clinical Biochemistry at the Chinese PLA General Hospital. TYP is the Director of the Department of Clinical Biochemistry at the Chinese PLA General Hospital, and a leader in the field of clinical laboratory examinations in China. He devotes his efforts in the field of diagnostics within the clinical laboratory.

## First co-authors

Jing Gao, Yong Wang and Zhennan Dong.

## Pre-publication history

The pre-publication history for this paper can be accessed here:

http://www.biomedcentral.com/1472-6947/12/58/prepub

## Supplementary Material

Additional file 1 PATIENT RESEARCH CONSENT FORM. Click here for file

Additional file 2 **Table S1.** Hispathologic diagnosis. Click here for file

Additional file 3 **Table S2.** Results of T test and U test of 57 biologic parameters. Click here for file

Additional file 4 **Table S3.** C statistics in ROC curves of 57 biologic parameters. Click here for file

Additional file 5 **Table S4.** Effects on the basic models by adding the 12 pre-select biological parameters. Click here for file
