# Supplementary material for: A novel differential diagnostic model based on multiple biological parameters for immunoglobulin A nephropathy
Source: BMC Med Inform Decis Mak. 2012 Jun 27;12:58. doi: 10.1186/1472-6947-12-58 (PMC3488968; doi:10.1186/1472-6947-12-58)
Supplement: Additional file 1 — PATIENT RESEARCH CONSENT FORM. [file 1472-6947-12-58-S1.doc]

**PATIENT RESEARCH CONSENT FORM**

**I, , agree to participate in a joint prospective research study titled “A novel differential diagnostic model based on multiple biological parameters for immunoglobulin A nephropathy” conducted by Dr. Yaping Tian, from Department of Clinical Biochemistry in Chinese PLA General Hospital, China. I understand that my participation is voluntary. I can refuse to participate or stop taking part at anytime without giving any reason, and without penalty or loss of benefits to which I am otherwise entitled. I can ask to have all of the information that can be identified as mine, returned to me, removed from the research records, or destroyed.**

**The purpose for this study is to find out and establish the possible model among clinical parameters for discriminating IgA nephropathy and non-IgA nephropathy.**

**If I volunteer to take part in this study, I will be asked to do the following things:**

1. **Allow the researchers to go through my medical records and obtain the data including blood biochemical indicators, the results of renal biopsy and histological diagnosis, and other information, including with or without hypertension, BMI, and so on.**
2. **Allow the researchers to analyze the data in my medical records, such as previous history, drug history, and family history.**
3. **Allow the researchers to draw my vein blood and test it for biological parameters.**

**I understand that my medical records including biochemical indicators are for further statistics analysis. I also understand that I do not have to offer the information of my medical records. I can still be in this study even if I choose to give part of information in medical records.**

**Only general clinical information such as gender, age, the type of kidney disease, and the data of general biochemical indicators are allowed to be public. Data involved in private, such HIV-AIDS, HBV, or HCV test results, are not allowed to be made available outside of hospital, are not allowed to be public, and only kept in local hospital.**

**No immediate benefit for me other than the satisfaction of contributing my medical information for research aimed at developing new diagnostic method for kidney nephropathy. I will not be compensated for participating in this study.**

**I understand that I am agreeing by my signature on this form to take part in this research project and understand that I will receive a signed copy of this consent form for my records.**

**Dr. Yaping Tian**

**_________________________ _______________________ __________**

# Name of Researcher Signature Date

**Telephone: ________________**

**Email: ____________________________**

**_________________________ _______________________ __________**

**Name of Participant Signature Date**

**Please sign both copies, keep one and return one to the researcher.**

**Additional questions or problems regarding your rights as a research participant should be addressed to The Chairperson, Institutional Review Board, PLA General Hospital, Fuxing Road 28, Beijing 100853, China; Telephone 0086-10-66939246.**
