# Supplementary material for: A novel differential diagnostic model based on multiple biological parameters for immunoglobulin A nephropathy
Source: BMC Med Inform Decis Mak. 2012 Jun 27;12:58. doi: 10.1186/1472-6947-12-58 (PMC3488968; doi:10.1186/1472-6947-12-58)
Supplement: Additional file 3 — Table S2. Results of T test and U test of 57 biologic parameters. [file 1472-6947-12-58-S3.doc]

**A novel differential diagnostic model based on multiple biological parameters for immunoglobulin A nephropathy**

**Supplement Table 2: Results of T test and U test of 57 biologic parameters.**

| **Index** | **Parameters** | **Mean±SD** | | **T test** | **Median (minimum, maximum)** | | **U test** |
| --- | --- | --- | --- | --- | --- | --- | --- |
|  |  | **IgAN** | **Non-IgAN** |  | **IgAN** | **Non-IgAN** |  |
| 1 | CEA | 1.8±1.4 | 2.0±1.5 | 0.393 | 1.4(0.2,8.8) | 1.5(0.2,7.6) | 0.446 |
| 2 | AFP | 2.4±1.1 | 2.9±1.8 | 0.066 | 2.1(0.6,5.4) | 2.3(0.6,9.2) | 0.227 |
| 3 | CA125 | 20.2±27.1 | 32.7±46.4 | 0.070 | 14.4(6.23,194.2) | 15.5(4.52,277.00) | 0.259 |
| 4 | CA199 | 12.0±8.7 | 18.8±20.3 | 0.016 | 10.5(0.6,47.2) | 12.6(0.6,130.2) | 0.046 |
| 5 | CA153 | 11.9±5.2 | 14.9±7.7 | 0.015 | 10.7(4.56,28.83) | 12.3(4.55,39.83) | 0.038 |
| 6 | CA724 | 2.3±2.6 | 2.9±3.2 | 0.277 | 1.2(0.72,15.4) | 1.3(0.76,14.24) | 0.244 |
| 7 | CYFRA21-1 | 2.8±1.6 | 3.1±1.3 | 0.237 | 2.5(0.87,10.33) | 3.0(1.07,6.05) | 0.069 |
| 8 | NSE | 11.2±3.3 | 11.0±3.6 | 0.810 | 10.5(6.13,20.16) | 10.5(1.00,23.22) | 0.959 |
| 9 | SCC | 1.5±0.8 | 1.4±1.0 | 0.605 | 1.4(0.4,4.7) | 1.2(0.1,5.9) | 0.173 |
| 10 | Glu | 5.0±0.7 | 5.2±0.9 | 0.292 | 4.9(3.85,7.84) | 5.0(4.00,10.07) | 0.216 |
| 11 | TP | 66.2±9.5 | 57.3±12.7 | 0.000 | 67.4(34,82) | 57.3(34.0,80.0) | 0.000 |
| 12 | ALB | 39.1±6.5 | 31.7±9.7 | 0.000 | 40.1(15.1,49.7) | 29.5(15.1,50.3) | 0.000 |
| 13 | UN | 6.6±3.1 | 6.1±3.4 | 0.389 | 6.0(3,22) | 4.96(2,20) | 0.048 |
| 14 | Cr | 102.8±71.6 | 93.2±59.8 | 0.422 | 84.2(36.6,540.6) | 75.7(39.4,374.8) | 0.118 |
| 15 | Ua | 394.9±110.7 | 358.4±116.8 | 0.081 | 391.5(161.7,704.3) | 342.8(121.7,703.0) | 0.073 |
| 16 | CH | 4.84±1.24 | 6.38±2.79 | 0..000 | 4.84(2.81,9.58) | 5.32(3.15,15.62) | 0.002 |
| 17 | TG | 1.7±1.1 | 2.1±1.0 | 0.073 | 1.4(0.57,6.86) | 2..01(0.49,4.47) | 0.013 |
| 18 | HDL | 1.1±0.3 | 1.3±0.4 | 0.060 | 1.1(0.68,1.88) | 1.2(0.62,2.53) | 0.136 |
| 19 | LDL | 2.98±1.00 | 4.14±2.23 | 0.000 | 2.88(1.14,6.81) | 3.36(1.47,11.47) | 0.003 |
| 20 | K | 4.0±0.5 | 4.1±0.4 | 0.841 | 4(3.05,5.3) | 4.0(3.08,5.94) | 0.748 |
| 21 | Na | 142.4±2.2 | 141.2±3.4 | 0.383 | 142.4(137.9,148.2) | 142.5(127.2,146.8) | 0.979 |
| 22 | Ca | 2.22±0.15 | 2.09±0.18 | 0.000 | 2.22(1.8,2.43) | 2.09(1.70,2.44) | 0.000 |
| 23 | Cl | 105.7±2.6 | 106.7±4.0 | 0.137 | 105.7(99.9,112.0) | 107.2(89.2,116.1) | 0.015 |
| 24 | P | 1.3±0.2 | 1.2±0.2 | 0.198 | 1.3(0.74,2.03) | 1.22(0.79,2.05) | 0.117 |
| 25 | Mg | 0.88±0.09 | 0.88±0.09 | 0.780 | 0.88(0.71,1.34) | 0.88(0.71,1.20) | 0.540 |
| 26 | CO2 | 25.9±2.6 | 26.1±2.6 | 0.760 | 26.2(17,30) | 26.3(18.5,31.7) | 0.864 |
| 27 | TB | 10.3±5.7 | 8.5±4.0 | 0.058 | 9(2,25) | 8.2(3,25) | 0.168 |
| 28 | DB | 3.1±1.8 | 2.4±1.3 | 0.013 | 2.6(0.5,8.5) | 2.1(0.5,7.3) | 0.029 |
| 29 | ALT | 20.6±13.6 | 24.1±16.9 | 0.212 | 16.5(7,73) | 17.7(6,87) | 0.132 |
| 30 | AST | 18.9±7.8 | 21.0±7.5 | 0.140 | 17.8(7.7,51.2) | 20.1(10.2,52.8) | 0.055 |
| 31 | LDH | 165.8±35.1 | 182.0±50.3 | 0.045 | 159.7(108.5,261.4) | 175.5(108.3,378.3) | 0.074 |
| 32 | CK | 128.8±165.0 | 96.9±68.8 | 0.162 | 89.2(27.9,1085.0) | 72(23.2,331.5) | 0.142 |
| 33 | GGT | 25.2±19.7 | 28.7±21.2 | 0.361 | 19.0(9.4,91.2) | 22.5(8.6,125.4) | 0.129 |
| 34 | ALP | 68.8±40.2 | 81.0±50.3 | 0.147 | 61(10.4,284.6) | 72(42.5,371.6) | 0.015 |
| 35 | INR | 0.95±0.08 | 0.96±0.09 | 0.772 | 0.94(0.82,1.13) | 0.95(0.76,1.30) | 0.848 |
| 36 | FIB | 3.63±1.00 | 5.00±2.60 | 0.000 | 3.45(2.19,7) | 4.38(2.28,18.00) | 0.000 |
| 37 | PT | 12.6±0.8 | 12.6±0.8 | 0.953 | 12.5(11.1,14.2) | 12.5(11.3,15.4) | 0.795 |
| 38 | PA | 110.8±16.3 | 109.8±14.9 | 0.708 | 111(82,144) | 109(67,140) | 0.889 |
| 39 | APTT | 36.7±4.2 | 36.7±5.1 | 0.942 | 36.8(29.2,50.6) | 36.1(28.7,56.7) | 0.644 |
| 40 | D2 | 0.70±1.26 | 1.46±2.99 | 0.069 | 0.4(0.09,8.66) | 0.53(0.08,20.00) | 0.019 |
| 41 | B_2_MG | 0.3±0.2 | 0.3±0.2 | 0.540 | 0.23(0.16,1.62) | 0.22(0.13,1.09) | 0.346 |
| 42 | sIgA | 331.3±103.9 | 241.5±102.3 | 0.000 | 329(175,620) | 225(67,600) | 0.000 |
| 43 | sIgG | 1018.5±307.0 | 858.2±352.7 | 0.009 | 974.5(350,1730) | 885(153,1890) | 0.020 |
| 44 | sIgE | 170.6±338.9 | 314.5±574.0 | 0.102 | 73.3(5.1,2300) | 115(7.9,3000) | 0.099 |
| 45 | sIgM | 107.7±55.2 | 125.7±71.2 | 0.121 | 99.2(23,333) | 105(30,327) | 0.243 |
| 46 | C3 | 111.2±22.7 | 119.0±25.4 | 0.078 | 108(71.4,185) | 117(51.2,196.0) | 0.061 |
| 47 | C4 | 26.1±5.9 | 27.3±8.9 | 0.360 | 25.4(14.8,41.5) | 26.0(6.9,56.1) | 0.492 |
| 48 | Prealbumin | 27.5±7.4 | 24.7±6.6 | 0.049 | 26.8(11.2,53.5) | 26.0(9.3,36.1) | 0.123 |
| 49 | RBC | 4.7±0.8 | 4.7±0.7 | 0.860 | 4.69(3.05,6.56) | 4.67(2.84,6.46) | 0.967 |
| 50 | HB | 135.4±27.7 | 139.6±22.9 | 0.372 | 137(6,180) | 141(87,194) | 0.629 |
| 51 | WBC | 8.8±12.5 | 7.4±2.4 | 0.382 | 7.07(4,101) | 6.97(3.42,15.93) | 0.891 |
| 52 | PLT | 242.1±65.0 | 235.9±70.7 | 0.616 | 239(116,509) | 222(138,506) | 0.329 |
| 53 | BMI | 24.9±3.7 | 24.8±4.0 | 0.827 | 24.6(16.4,35.6) | 24.6(17.3,37.2) | 0.789 |
| 54 | HP | \ | \ |  | 0(0,1) | 0(0,1) | 0.674 |
| 55 | Gender | \ | \ |  | 1(1,2) | 1(1,2) | 0.098 |
| 56 | Age | 35.6±12.4 | 39.6±15.3 | 0.110 | 34(10，67) | 41(11,68) | 0.123 |
| 57 | Manifestation | \ | \ |  | 1(0,2) | 2(0,2) | 0.000 |

Significance level: P<0.05
