# Supplementary material for: A novel differential diagnostic model based on multiple biological parameters for immunoglobulin A nephropathy
Source: BMC Med Inform Decis Mak. 2012 Jun 27;12:58. doi: 10.1186/1472-6947-12-58 (PMC3488968; doi:10.1186/1472-6947-12-58)
Supplement: Additional file 4 — Table S3. C statistics in ROC curves of 57 biologic parameters. [file 1472-6947-12-58-S4.doc]

**A novel differential diagnostic model based on multiple biological parameters for immunoglobulin A nephropathy**

**Supplement Table 3: C statistics in ROC curves of 57 biologic parameters.**

| **Index** | **Parameter** | **AUC**  **(IgA group As state variable)** | **95%Confidence Interval** | | **P value** |
| --- | --- | --- | --- | --- | --- |
|  |  |  | **Lower Bound** | **Upper Bound** |  |
| 1 | CEA ^b^ | 0.540 | 0.437 | 0.643 | 0.446 |
| 2 | AFP ^b^ | 0.546 | 0.461 | 0.666 | 0.227 |
| 3 | CA125 ^b^ | 0.560 | 0.457 | 0.662 | 0.259 |
| 4 | CA199 ^b^ | 0.605 | 0.505 | 0.706 | 0.046 |
| 5 | CA153 ^b^ | 0.609 | 0.509 | 0.709 | 0.038 |
| 6 | CA724 ^b^ | 0.561 | 0.459 | 0.664 | 0.244 |
| 7 | CYFRA21-1 ^b^ | 0.596 | 0.494 | 0.697 | 0.069 |
| 8 | NSE ^b^ | 0.503 | 0.399 | 0.607 | 0.959 |
| 9 | SCC ^a^ | 0.572 | 0.470 | 0.674 | 0.174 |
| 10 | Glu ^b^ | 0.559 | 0.457 | 0.662 | 0.261 |
| 11 | TP ^a^ | 0.703 | 0.611 | 0.796 | 0.000 |
| 12 | ALB ^a^ | 0.727 | 0.636 | 0.819 | 0.000 |
| 13 | UN ^a^ | 0.604 | 0.503 | 0.706 | 0.048 |
| 14 | Cr ^a^ | 0.583 | 0.481 | 0.684 | 0.118 |
| 15 | Ua ^a^ | 0.595 | 0.493 | 0.696 | 0.073 |
| 16 | CH ^b^ | 0.667 | 0.572 | 0.763 | 0.002 |
| 17 | TG ^b^ | 0.631 | 0.532 | 0.731 | 0.013 |
| 18 | HDL ^b^ | 0.579 | 0.477 | 0.680 | 0.136 |
| 19 | LDL ^b^ | 0.657 | 0.560 | 0.753 | 0.003 |
| 20 | K ^b^ | 0.517 | 0.413 | 0.621 | 0.748 |
| 21 | Na ^a^ | 0.501 | 0.398 | 0.605 | 0.979 |
| 22 | Ca ^a^ | 0.718 | 0.625 | 0.810 | 0.000 |
| 23 | Cl ^b^ | 0.628 | 0.528 | 0.729 | 0.015 |
| 24 | P ^a^ | 0.583 | 0.481 | 0.686 | 0.117 |
| 25 | Mg ^a^ | 0.532 | 0.429 | 0.636 | 0.540 |
| 26 | CO2 ^b^ | 0.509 | 0.405 | 0.613 | 0.864 |
| 27 | TB ^a^ | 0.573 | 0.468 | 0.677 | 0.168 |
| 28 | DB ^a^ | 0.615 | 0.514 | 0.717 | 0.029 |
| 29 | ALT ^b^ | 0.580 | 0.477 | 0.682 | 0.132 |
| 30 | AST ^b^ | 0.601 | 0.500 | 0.702 | 0.055 |
| 31 | LDH ^b^ | 0.595 | 0.493 | 0.696 | 0.074 |
| 32 | CK ^a^ | 0.578 | 0.475 | 0.680 | 0.142 |
| 33 | GGT ^b^ | 0.580 | 0.478 | 0.682 | 0.129 |
| 34 | ALP ^b^ | 0.628 | 0.528 | 0.729 | 0.015 |
| 35 | INR ^b^ | 0.510 | 0.405 | 0.615 | 0.848 |
| 36 | FIB ^b^ | 0.712 | 0.621 | 0.804 | 0.000 |
| 37 | PT ^a^ | 0.514 | 0.409 | 0.618 | 0.795 |
| 38 | PA ^a^ | 0.507 | 0.402 | 0.613 | 0.889 |
| 39 | APTT ^a^ | 0.524 | 0.421 | 0.628 | 0.644 |
| 40 | D2 ^b^ | 0.626 | 0.525 | 0.727 | 0.019 |
| 41 | B_2_MG ^a^ | 0.553 | 0.444 | 0.661 | 0.346 |
| 42 | sIgA ^a^ | 0.756 | 0.670 | 0.842 | 0.000 |
| 43 | sIgG ^a^ | 0.623 | 0.524 | 0.722 | 0.020 |
| 44 | sIgE ^b^ | 0.587 | 0.485 | 0.690 | 0.099 |
| 45 | sIgM ^b^ | 0.562 | 0.458 | 0.665 | 0.243 |
| 46 | C3 ^b^ | 0.599 | 0.498 | 0.700 | 0.061 |
| 47 | C4 ^b^ | 0.536 | 0.433 | 0.639 | 0.492 |
| 48 | Prealbumin ^a^ | 0.590 | 0.478 | 0.702 | 0.123 |
| 49 | RBC ^b^ | 0.502 | 0.398 | 0.606 | 0.967 |
| 50 | HB ^b^ | 0.525 | 0.422 | 0.629 | 0.629 |
| 51 | WBC ^a^ | 0.507 | 0.403 | 0.611 | 0.891 |
| 52 | PLT ^a^ | 0.551 | 0.448 | 0.655 | 0.329 |
| 53 | BMI ^a^ | 0.514 | 0.410 | 0.618 | 0.789 |
| 54 | HP ^a^ | 0.519 | 0.416 | 0.622 | 0.716 |
| 55 | Gender ^b^ | 0.571 | 0.454 | 0.688 | 0.242 |
| 56 | Age ^b^ | 0.595 | 0.479 | 0.711 | 0.116 |
| 57 | Manifestation ^b^ | 0.717 | 0.624 | 0.810 | 0.000 |

Significance level: P<0.05; ^a^: IgAN group as state variable; ^b^: non-IgAN group as state variable.
