# Supplementary material for: A novel differential diagnostic model based on multiple biological parameters for immunoglobulin A nephropathy
Source: BMC Med Inform Decis Mak. 2012 Jun 27;12:58. doi: 10.1186/1472-6947-12-58 (PMC3488968; doi:10.1186/1472-6947-12-58)
Supplement: Additional file 5 — Table S4. Effects on the basic models by adding the 12 pre-select biological parameters. [file 1472-6947-12-58-S5.doc]

**A novel differential diagnostic model based on multiple biological parameters for immunoglobulin A nephropathy**

**Supplement table 4: Effects on the basic models by adding the 12 pre-select biological parameters.**

| **Order** | **Parameter** | **Logistic regression** | | | **Discriminant analysis** | | |
| --- | --- | --- | --- | --- | --- | --- | --- |
|  |  | **Sensitivity** | **Specificity** | **Accuracy** | **Sensitivity** | **Specificity** | **Accuracy** |
| 0 | Gender +Manifestation | 0.893 | 0.586 | 0.740 | 0.893 | 0.586 | 0.740 |
| 1 | sIgA | 0.839 | 0.736 | 0.788 | 0.871 | 0.690 | 0.781 |
| 2 | sIgA+ALB | 0.839 | 0.736 | 0.788 | 0.871 | 0.690 | 0.781 |
| 3 | sIgA+ALB+FIB | 0.871 | 0.759 | 0.815 | 0.892 | 0.736 | 0.814 |
| 4 | sIgA+ALB+FIB +CH | 0.860 | 0.770 | 0.815 | 0.892 | 0.724 | 0.808 |
| 5 | sIgA+ALB+FIB +CH+TG | 0.806 | 0.759 | 0.783 | 0.849 | 0.736 | 0.793 |
| 6 | sIgA+ALB+FIB +CH+TG+ALP | 0.828 | 0.770 | 0.799 | 0.882 | 0.736 | 0.809 |
| 7 | sIgA+ALB+FIB +CH+TG+ALP +D2 | 0.828 | 0.770 | 0.799 | 0.882 | 0.747 | 0.815 |
| 8 | sIgA+ALB+FIB +CH+TG+ALP +D2+sIgG | 0.796 | 0.793 | 0.7945 | 0.839 | 0.759 | 0.799 |
| 9 | sIgA+ALB+FIB +CH+TG+ALP +D2+sIgG+DB | 0.806 | 0.793 | 0.800 | 0.849 | 0.759 | 0.804 |
| 10 | sIgA+ALB+FIB +CH+TG+ALP +D2+sIgG+DB+CA153 | 0.806 | 0.793 | 0.800 | 0.849 | 0.724 | 0.787 |
| 11 | sIgA+ALB+FIB +CH+TG+ALP +D2+sIgG+DB+CA153+CA199 | 0.806 | 0.793 | 0.800 | 0.849 | 0.759 | 0.804 |
| 12 | sIgA+ALB+FIB +CH+TG+ALP +D2+sIgG+DB+CA153+CA199+sUN | 0.817 | 0.782 | 0.800 | 0.849 | 0.759 | 0.804 |
